# Cross‐hierarchical plasticity of corticofugal projections to dLGN after neonatal monocular enucleation

**DOI:** 10.1002/cne.25304

**Published:** 2022-02-15

**Authors:** Chrysoula Giasafaki, Eleanor Grant, Anna Hoerder‐Suabedissen, Shuichi Hayashi, Sheena Lee, Zoltán Molnár

**Affiliations:** ^1^ Department of Physiology Anatomy and Genetics Oxford UK; ^2^ Instituto de Neurociencias de Alicante CSIC‐UMH, Av. Ramon y Cajal s/n 03550 San Juan de Alicante Alicante Spain; ^3^ Department of Anatomy Kawasaki Medical School Kurashiki Okayama 701‐0192 Japan

**Keywords:** cortex, cross hierarchical cortico‐thalamic plasticity, layer 5, Rbp4‐cre, thalamus

## Abstract

Perception is the result of interactions between the sensory periphery, thalamus, and cerebral cortex. Inputs from the retina project to the first‐order dorsal lateral geniculate nucleus (dLGN), which projects to the primary visual cortex (V1). In return, the cortex innervates the thalamus. While layer 6 projections innervate all thalamic nuclei, cortical layer 5 neurons selectively project to the higher order lateral posterior nucleus (LP) and not to dLGN. It has been demonstrated that a subpopulation of layer 5 (Rbp4‐Cre+) projections rewires to dLGN after monocular or binocular enucleation in young postnatal mice. However, the exact cortical regional origin of these projections was not fully determined, and it remained unclear whether these changes persisted into adulthood. In this study, we report gene expression changes observed in the dLGN after monocular enucleation at birth using microarray, qPCR at P6, and in situ hybridization at P8. We report that genes that are normally enriched in dLGN, but not LP during development are preferentially downregulated in dLGN following monocular enucleation. Comparisons with developmental gene expression patters in dLGN suggest more immature and delayed gene expression in enucleated dLGN. Combined tracing and immuno‐histochemical analysis revealed that the induced layer 5 fibers that innervate enucleated dLGN originate from putative primary visual cortex and they retain increased VGluT1+ synapse formation into adulthood. Our results indicate a new form of plasticity when layer 5 driver input takes over the innervation of an originally first‐order thalamic nucleus after early sensory deficit.

## Graphical abstract

In this study, the effect of monocular enucleation (MoE) in the development of the first‐order visual thalamic nucleus, dLGN, was examined. We reported by microarray analysis a transcriptionally immature dLGN after early MoE. Additionally, focusing on layer 5 axonal projections, we demonstrated by viral tracing that layer 5 fibers in the enucleated dLGN originate from the primary visual cortex and not from the primary somatosensory cortex. Finally, we showed that layer 5 axons reorganize while innervating the enucleated dLGN and form aberrant synapses that persist until adulthood, indicating the presence of a permanent cross‐hierarchical cortico‐thalamic plasticity.

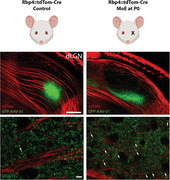

## 1 INTRODUCTION

The thalamus has been widely considered as the relay center for sensory information to the cortex, but it also has an essential role in the regulation of fundamental brain processes, including sleep, alertness, consciousness, and cognition, via various distinct nuclei (Jones, 1985). Almost all sensory information reaches our cerebral cortex via the thalamus and in return, the cerebral cortex sends projections to the thalamus to regulate this input. The thalamic nuclei that receive direct sensory input are referred to as first‐order nuclei, whereas those receiving their input from the cerebral cortex, and relaying this back to the cortex, are referred to as higher order thalamic nuclei (Sherman & Guillery, 1998). Understanding plasticity of thalamocortical pathways and their higher cognitive functions is not possible without considering the broader thalamo‐cortical (TC) network. Recent evidence suggests a direct role of the thalamus in generating complex cognitive functions through cortico‐thalamo‐cortical connections (Shepherd & Yamawaki, 2021). For instance, optogenetic silencing of the associative thalamus—but not the sensory relays (sensory or first‐order nuclei)—during a working memory task severely impacts task performance (Guo et al., 2017; Schmitt et al., 2017).

The higher order thalamic nuclei do not receive direct sensory input from sensory organs; their driver input originates from the layer 5 and some layer 6b projection neurons (Grant et al., 2012, 2016; Hoerder‐Suabedissen et al., 2019). Specifically in the visual system, inputs from the retina project to the first‐order dorsal lateral geniculate nucleus (dLGN) of the thalamus, which projects to the primary visual cortex (V1). While layer 6 projections innervate all thalamic nuclei, cortical layer 5 neurons selectively project to the higher order lateral posterior nucleus (LP) of the thalamus and do not normally innervate dLGN.

While the plasticity of the thalamocortical projections has been widely appreciated after sensory deprivation and various manipulation studies (Molnár, 1998; Pallas & Sur, 1993), the changes in the corticothalamic connectivity have received less attention. The introduction of cell‐subtype specific Cre mouse lines opened possibilities for the selective monitoring and manipulation of subsets of corticothalamic projections (Frangeul et al., 2016; Grant et al., 2012, 2016; Hoerder‐Suabedissen et al., 2019; Korrell et al., 2019; Krone et al., 2021). Using layer 5 reporter gene expressing mice, it has been demonstrated that layer 5b (Rbp4‐Cre+) projections rewire to innervate dLGN after monocular (Grant et al., 2016) or binocular enucleation (Frangeul et al., 2016) in young postnatal mice. These studies introduced a new form of plasticity, where the layer 5 driver input of the cortex innervates a first‐order thalamic nucleus that lost its driver input from the sensory periphery. Both models showed this form of plasticity, although in monocular enucleation (MoE) there is some residual retinal input to the “enucleated dLGN” (the dLGN contralateral to the enucleation) from the ipsilateral eye and this may target a larger section of the dLGN in absence of the contralateral input. However, the exact cortical areal origin of these projections was not fully determined, and it remained unclear whether these changes in young postnatal brains persist into adulthood.

In this study, we analyzed the gene expression changes observed in the P6 dLGN after MoE at birth using microarrays and confirmed the results with quantitative PCR (qPCR). We report that genes enriched in dLGN, but not LP, in normally developing brains, are preferentially downregulated following MoE, and enucleated dLGN is more immature in its gene expression. Moreover, we present data on the distribution of the altered gene expression of four selected genes with additional in situ hybridization data. Two of these additionally change their expression pattern in contralateral ventral LGN after MoE. Furthermore, *Efna5*, the expression of which is usually undetectable in LP, increased its expression in this higher order thalamic nucleus after enucleation.

Additionally, we demonstrate that the cross‐hierarchical rewiring of cortical layer 5 afferents to the enucleated dLGN— the dLGN contralateral to the enucleation—persists into adulthood, and that the aberrant layer 5 fibers in dLGN do not derive from cortical areas serving other sensory modalities. Primary visual cortex‐derived layer 5 axons in enucleated dLGN retain increased VGluT1+ synapse formation into adulthood. Our results have two major implications: (i) that layer 5 driver input that takes over the innervation of dLGN after early visual sensory deficit is maintained to adulthood and (ii) this layer 5 input originates from the putative primary visual cortex.

## 2 METHODS

### 2.1 Animals

All experiments were performed in accordance with U.K. Home Office regulations and local ethical review under valid Animals (Scientific Procedures) Act personal and project licenses.

C57/BL6 wild type (WT) mice were obtained from Charles River (UKC57/Bl6). Females were time mated for 12 h overnight and 12:00 p.m. the next day was designated as E0.5. Day of birth was designated P0 and only litters that were born between E18.5 and E19.5 were used. MoE was performed at P0 as described previously (Grant et al., 2016). At P6, the pups were killed by cervical dislocation and processed immediately, keeping time between sacrifice and protection of the micro‐dissected dLGN pieces in RNALater (R0901‐250 ml; Sigma Aldrich) minimal. Brains were dissected in RNase‐free conditions, embedded in low‐melting point agarose (Sigma‐Aldrich) and cut to 200 μm coronal slices using a vibrating microtome (Leica VT1000S). Sectioning and subsequent dissection took place under sterile and RNase free conditions in artificial cerebrospinal fluid (aCSF, made up with DEPC‐water) containing 126 mM NaCl, 26.4 mM NaHCO_3_, 3 mM KCl, 2 mM CaCl_2_, 2 mM MgSO_4_, 1.2 mM H_2_NaPO_4_, and 10 mM glucose. The dLGN was immediately manually microdissected under visual guidance in order to avoid unintentional dissection of adjacent areas, such as IGL, vLGN, VMP, or LP and tissue pieces were put into 500 μl of RNALater. Three pieces of dLGN were collected per hemisphere per brain. Samples were stored at −20°C overnight to ensure full RNALater penetration of the tissue, before being stored at −80°C until RNA extraction. For the microarray, four replicates were used. Eight pups were used per replicate from one or two litters per replicate (three replicates contained pups from two litters, the remaining replicate consisted of one litter). No litter was used in more than one replicate. For the real‐time quantitative PCR (RT‐qPCR), two replicates were derived from pooled microarray replicates, and a further two replicates were newly generated, with each replicate containing dLGN fragments from 16 pups derived from four litters. No litter was used in more than one replicate. To confirm gene expression alterations using in situ hybridization, and further validate the MoE model, Tg(Rbp4‐cre)KL100Gsat/Mmucd (Rbp4‐Cre) mice were crossed to B6;129S6‐Gt(ROSA)26Sortm14(CAG‐tdTomato)Hze/J (Ai14) mice to generate a tdTomato labeled subset of L5 neurons. MoE was performed as previously described (Grant et al., 2016). Following enucleation, P6 mice were killed by cervical dislocation and brains were dissected out in RNAse‐free conditions. Dissected brains were embedded in OCT compound (Tissue Tek) on dry ice and stored at −80°C.

### 2.2 Microarray

Samples contributing to the same replicate were pooled. Four replicates were used. Each replicate contained tissue from eight mice (due to the amount of tissue dissected from each mouse, tissue from eight mice had to be pooled together to form one replicate). The eight mice in each replicate were taken from one or two litters. No litter contributed to more than one replicate. RNA extraction was performed using the Qiagen RNeasy Micro Kit (Qiagen Ltd, Manchester UK, 74004) according to the manufacturer's instructions, including the DNaseI step to remove contaminating DNA. RNA concentration was assessed by Nanodrop (Thermo Scientific, DE, USA) and quality was assessed by Agilent 2100 Bioanalyser Nano‐Chip (Agilent Technologies Inc, CA, USA). Only replicates with RNA Integrity Numbers (RIN) of 8 or above were used for the microarray. Starting material of 40 ng RNA was reverse transcribed and linearly amplified using the NuGen Ovation Pico WTA System V2 (NuGen Technologies Inc, CA, USA, 3302–12). The amplified, double‐stranded cDNA was transformed into single strand sense cDNA that was then chemically and enzymatically fragmented to produce strands of 50–100 bps in length. The sample was run on an Agilent Bioanalyser NanoChip (Agilent) to confirm successful fragmentation. The strands were then labeled by biotin‐labeled nucleotide to the 3‐hyrdoxyl end. The fragmentation and biotin process were performed using the Encore Biotin Module (NuGen, 4200–12). Fragmented, labeled single strand sense cDNA was hybridized to the Affymetrix GeneChip Mouse 1.0 ST Array chip (Affymetrix UK Ltd, High Wycombe, UK), and the hybridized chips scanned on the Affymetrix Gene Chip Scanner and GeneChip Operating System (Affymetrix).

### 2.3 Microarray statistical analysis

Data processing was performed with GeneChip® Command Console® Software (AGCC) and normalized using the Robust Multichip Average (RMA) package in GeneSpring GX 12.6.1 (Irizarry et al., 2003a, 2003b). The gene expression values were compared between control and enucleated dLGN using Limma Analysis (Linear Models for Microarray Data; Smyth, 2004) in GeneSpring GX 12.6.1 (Agilent Technologies, Germany), with a cut‐off of 1.3‐fold change or greater. A paired design and Benjamini–Hochberg multiple testing correction (Benjamini & Hochberg, 1995) were used. Gene expression values were also compared using a two‐way ANOVA followed by a paired *t*‐test. The intersection of both gene lists was used to generate a final list using the Partek Genomics Suite (Partek Inc. Saint Louis, MO). To analyze functional enrichment in the list of genes which are differentially expressed genes, we used DAVID (Database for Annotation, Visualization and Integrated Discovery; Huang da et al., 2009a, 2009b) on the Limma analysis generated list.

### 2.4 Real‐time quantitative PCR

To validate the genes identified as differentially expressed by Limma analysis, real‐time qPCR was performed. RNA was extracted and quality assessed as described above. Only replicates with RIN of 5 or above were used for the qPCR. First strand cDNA was synthesized using the Qiagen Reverse Transcription kit (Qiagen 330401), including the DNase step. Note that 200 ng of RNA starting material was used. A known concentration of exogenous RNA was spiked into each sample for confirmation of reverse transcription efficiency after the qPCR run. Commercially available primers from Qiagen were used and as such primer sequences are not disclosed. Primers were designed to have uniform primer length, GC content, and annealing temperature. Primer specificity was confirmed experimentally by performing dissociation curve analysis on the PCR products after each qPCR run. The dissociation curve of our samples showed one thermal transition in fluorescence thus confirming that there were no none‐specific targets of the primers. qPCR was performed using Qiagen RT^2^ SYBR Green ROX qPCR Master mix containing HotStart DNA Taq polymerase, PCR buffer, dNTP mix, SYBR Green dye, and ROX passive reference dye (Qiagen 330523). The qPCR was run on the Stratagene Mx3005P (Agilent). Each biological replicate was run three times to provide technical replicates. No technical replicates were deemed as outliers and as such, all technical replicates for each biological sample were treated collectively. The exogenous RNA spike confirmed that reverse transcription was efficiently performed and was of uniform efficiency across all samples. A specific genomic DNA primer confirmed the lack of genomic DNA contamination in all samples. A spiked genomic DNA well confirmed the efficiency of the PCR amplification for each sample. Housekeeping genes were used as references to normalize the cycle number at which the threshold is crossed (Ct) values to control for variance in efficiency of RNA isolation or reverse transcription across samples or qPCR runs. We chose reference genes that were stably expressed in mouse brain tissue at early postnatal ages: *Pgk1* and *Tfrc* (Boda et al., 2009), and *Hprt1* (Vandesompele et al., 2002).

### 2.5 In situ hybridization

OCT embedded P8 brains were sectioned coronally to 20 μm on a cryostat (Leica, Jung CM3000) and mounted on 1.0 mm SuperFrost Ultra Plus slides (Thermo Scientific™). In order to validate the gene expression changes of some of the genes included in the RT‐qPCR analysis in situ hybridization was performed (*n *= 3 replicates for each probe). Riboprobes for orthodenticle homeobox 2 (Otx2), EphA5, Calb2 and Cbln2 were synthesized as previously described (Hoerder‐Saubedissen et al., 2009; Oeschger et al., 2012), using the following primers: Calb2 forward primer: 5′*‐GATGCTGACGGAAATGGG*; Calb2 reverse primer: 5′*‐CCCTACCAGCCACCCTCT*; Cbln2 forward primer: 5′*‐CAGCTTCCACGTGGTCAA*; Cbln2 reverse primer: 5′*‐AGCCCCCAGCATGAAAAC*; Otx2 forward primer: 5′‐*TCCAGCTCGGGAAGTGAG*; Otx2 reverse primer: 5′‐*AGGCCATGACCTTCCCTC*; Efna5 forward primer: 5′*‐CGTCTACTGGAACAGCAGCA*; Efna5 reverse primer: *5′‐TGACATCTGCCAAAAACCAA*.

In situ hybridization was performed as previously described (Hoerder‐Saubedissen et al., 2009; Oeschger et al., 2012). Briefly, digoxigenin (DIG) labeled RNA probes against *Otx2* (500 ng), *EphA5* (400 ng), *Calb2* (500 ng), and *Cbln2* (600 ng), were diluted in hybridization buffer (50% formamide, 10 mM Tris [pH 7.6], 200 μg/ml *Escherichia coli* transfer RNA, 1 × Denhardt's solution, 10% dextran sulfate, 600 mM NaCl, 0.25% sodium dodecyl sulfate [SDS], 1 mM ethylenediaminetetraacetic acid [EDTA]) and applied on the sections for overnight incubation at 70°C in a humidified chamber. Following probe incubation, slides were stringently washed in sodium citrate saline followed by further washes in TBS [100 mM NaCl, 100 mM Tris‐Cl (pH7.5)]. Sections were blocked with 0.5% Boehringer Blocking Reagent (Roche) in TBS for 1 h at room temperature followed by incubation with alkaline phosphatase (AP) anti‐DIG antibody (Roche, 1:2000 in blocking solution) at 4°C overnight. Following the antibody incubation, sections were washed with NTM (100 mM Tris [pH 9.5], 100 mM NaCl, 50 mM MgCl_2_) prestaining buffer and then incubated at 4°C with a staining buffer containing NBT (nitro blue tetrazolium)/BCIP (5‐bromo‐4‐chloro‐3‐indolyl‐phosphate) (Roche) in humidified chamber and monitored for the next 16–48 h, depending on each probe, for development of the desired color reaction. For fluorescent color reaction, Fast Red TR/Naphthol AS‐MX Tablets (Sigma‐Aldrich) were used for detection of the AP anti‐DIG antibody. Note that 1× Tris (pH 8.2) was used as a prestaining buffer and for dilution of the tablets. Incubation with the fast red staining buffer lasted overnight at 4°C. Fast red incubated sections were counterstained with DAPI and mounted in FluorSave (Millipore).

### 2.6 Unilateral viral injections on targeted brain regions

To trace the axonal projections of layer 5 cells to subcortical targets, especially to thalamus and superior colliculus, in Rbp4‐Cre::tdTomato control and monocularly enucleated mice at P0, we performed unilateral viral injections of a Cre‐independent GFP adeno‐associated virus (AAV) targeting cortical layer 5 contralateral to the enucleation, therefore targeting the thalamus with the dLGN with reduced retinal input, referred to as enucleated dLGN. Adult mice (age range 8–10 weeks) were deeply anesthetized with isoflurane and placed in a stereotaxic frame. After midline skin incision, a unilateral craniotomy was performed over V1 or S1. Using a calibrated glass micropipette, mice were injected into the right hemisphere with either 100 nl of AAV2‐CAGGS‐Arch‐GFP (University of North Carolina Vector Core, Ed Boyden repository) Cre independent virus in monocular sector of V1 (V1M; *n* = 3 for each condition), or 200 nL of the same virus in the barrel field area of S1 (*n* = 3 for MoE animals). Injection was over the course of 1 min, and micropipette was left in place for another 5 min to reduce reflux of virus. After retraction of the micropipette, the skin was sutured and animals were allowed to recover in a heated recovery chamber, before being returned to their home cage. Appropriate analgesia was provided during and after the surgery. We chose to inject the right hemisphere of the mice as we enucleated their left eye thus depriving of retinal input the right hemisphere. At 3–4 weeks after the surgery, when GFP was expressed, mice were perfused.

### 2.7 Image acquisition and processing

Fluorescent and bright field microscope images were obtained using a Leica epifluorescence microscope (DMR) with a Leica DC500 camera. Fast red‐stained slides were imaged on an inverted confocal microscope (Olympus FV1200). Images were contrast adjusted on ImageJ software, and final figures generated using Adobe Photoshop. Schematic illustrations were made using Adobe Illustrator.

### 2.8 Data analysis

For analyzing the imaging data acquired, all images were brightness and contrast adjusted, their background was subtracted and they were auto‐thresholded by using the respective plugins on ImageJ software. For measurement of the thickness of the tdTom+ dorsal axon bundle, the length of the bundle on the dorsal part of dLGN was quantified using ImageJ. Colocalization of the tdTomato+ and VGluT1+ boutons formed in control and enucleated dLGN was quantified from confocal stack images of the upper lateral part of dLGN and compared using a two‐tailed, unpaired, Student's *t*‐test (*n* = 3 brains, at least three medial sections per brain were measured). For the comparison of control and enucleated dLGN size and the thickness of the tdTom+ dorsal axon bundle, analysis was done by using a two‐tailed, unpaired, Student's *t*‐test (*n* = 9 independent brain samples). For measuring the signal intensity of Otx2 expression in the fluorescent in situ hybridization experiments, the integrated density was quantified on ImageJ in control and enucleated dLGN and analyzed using a two‐tailed, paired, Student´s *t*‐test (*n* = 3 brains, three medial sections per brain were measured). As expression of Otx2 was very specific, signal intensity of individual ROIs with FastRed staining in the dLGN was quantified. Cell density of Otx2 positive cells in control and enucleated dLGN was quantified by colocalization of FastRed+ cells with DAPI, and compared using a two‐tailed, paired Student´s *t*‐test (*n* = 3 brains, three medial sections per brain were measured). Statistical analysis and graph generation were performed on GraphPad Prism 8. Differences were considered significant when *p *< .05.

## 3 RESULTS

### 3.1 Gene expression pattern in the deafferented dLGN using microarray

Following MoE at P0, gene expression in the control and the enucleated dLGN at P6 was compared using Affymetrix GeneChip Mouse 1.0 ST microarrays. All samples were processed at the same time. Quality control by the Expression Console™ 1.2.0.20 (Affymetrix) identified sample (5c) from the initial microarray run as faulty. That sample was run again, and all measures of quality control confirmed that the separate run did not affect the results of the sample. The Pearson correlation heat map demonstrates that ipsilateral samples are more similar to one another, and contralateral samples are more similar to one another. To avoid cross‐contamination with other adjacent areas, such as the IGL, vLGN, ventral‐posteromedial nucleus (VPM), or LP, microarray data have been validated for their purity comparing gene expression between dLGN and nearby nuclei. We used previous publications that selectively explored gene expression differences between vLGN/IGL and dLGN (Su et al., 2011). We explored the expression of vLGN/IGL‐specific genes described by Su and colleagues (including *Sema3a, Sema3c, slit2, slit3, wnt5a, thbs4*) and none of these were represented in our list. We have also searched for IGL (*Penk, Rspo2*), vLGN (*Slc17a7, Neurod6, Htr1a, Dlx5*), reticular nucleus (*Pvalb*), and LP (*Necab1, Gpr4, Slc17a6*) specific genes identified in the single‐cell RNA sequencing data of the mouse dLGN and adjacent vLGN, IGL, and LP from Bakken et al. (2021) and compared them with our microarray dLGN data set, but again we have not found any increase of the specific genes that would indicate the inclusion of the above areas in our data. These comparisons suggest relative purity of our dLGN dissection. Additionally, we have compared our data set with the microarray data of postanatally bilaterally enucleated dLGN presented by Frangeul et al. (2016) and the vast majority of genes identified in our microarray also appeared in their microarray data, further confirming validity and consistency of our results with other similar studies.

51 genes were differentially expressed between the control and the enucleated dLGN, when using a fold‐change cut‐off of 1.3. Of these, 33 genes were downregulated and 14 were upregulated in the enucleated dLGN compared to the control dLGN. This number of genes is similar to other microarray studies which have been based on sensory deprivation or altered input to thalamic nuclei (Brooks et al., 2013; Horng et al., 2009; Majdan & Shatz, 2006). The greatest change in gene expression was of RIKEN cDNA E530001K10, which had a relative expression of 0.3031 (fold change of −3.2987) in the enucleated dLGN (Table 1).

**TABLE 1:** A list of the genes with differential expression in the enucleated dLGN compared to the control dLGN at P6 using Limma with a fold change cut off value of > 1.3 or < 0.77. Moderated *p* value is based on the moderated *t*‐statistic generated by Limma analysis. Downregulated genes are shaded blue. Upregulated genes are shaded red

Gene symbol	Gene name	Fold‐change	Moderated *p* value
	RIKEN cDNA E530001K10 gene	0.30	.000000003
Hmcn1	hemicentin 1	0.50	.000000098
Shc3	src homology 2 domain‐containing transforming protein C3	0.57	.000000034
Kcnk9	potassium channel, subfamily K, member 9	0.57	.000000039
Dgkk	diacylglycerol kinase kappa	0.61	.000008096
Hcrtr2	hypocretin (orexin) receptor 2	0.66	.000000509
Fam19a4	Family with sequence similarity 19, member A4	0.66	.000011353
Osbp13	Oxysterol binding protein‐like 3	0.66	.000000539
Dusp4	Dual specificity phosphatase 4	0.67	.000014830
Gjd2	Gap junction protein, delta 2	0.68	.000000046
Vsnl1	Visinin‐like 1	0.68	.000006828
Tacstd2	Tumor‐associated calcium signal transducer 2	0.7	.000015436
Myot	Myotilin	0.7	.000000174
Sncg	Synuclein, gamma	0.7	.000003186
Spred2	Sprouty‐related, EVH1 domain containing 2	0.7	.000000123
6530302D11Rik	RIKEN cDNA 6530402D11 gene	0.7	.000059895
Moxd1	Monooxygenase, DBH‐like 1	0.7	.000019658
Kcnn3	Potassium intermediate/small conductance calcium‐activated channel, subfamily N, member 3	0.71	.000027230
Shisa6	Shisa homologue 6 (Xenopus laevis)	0.74	.000001553
Chrm2	Cholinergic receptor, muscarinic 2, cardiac	0.74	.000107831
Adra1d	Adrenergic receptor, alpha 1d	0.75	.000006886
Fos	FBJ osteosarcoma oncogene	0.75	.000010900
Frem3	Fras1 related extracellular matrix protein 3	0.75	.000003221
Ptgr1	Prostaglandin reductase 1	0.75	.000275161
Hmgn5	High‐mobility group nucleosome binding domain 5	0.75	.000043902
Gfra1	Glial cell line derived neurotrophic factor family receptor alpha 1	0.76	.000025735
Dgkg	Diacylglycerol kinase, gamma	0.76	.000027844
Igf1	Insulin‐like growth factor 1	0.76	.000001483
Pdlim5	PDZ and LIM domain 5	0.76	.000108262
Col6a5	Collagen, type VI, domain 5	0.76	.000095315
Synm	Synemin, intermediate filament protein	0.76	.000130799
Chst2	Carbohydrate sulfotransferase 2	0.77	.000001235
Calb2	Calbindin 2	0.77	.000041262
Adamts3	A disintegrin‐like and metallopeptidase (reprolysin type) with thrombospondin type 1 motif, 3	0.77	.000026403
Timp4	Tissue inhibitor of metalloproteinase 4	0.77	.000058683
Egr2	Early growth response 2	0.78	.000181961
Mxd4	Max dimerization protein 4	1.3	.000002559
Siah3	Seven in absentia homologue 3 (Drosophila)	1.3	.000057871
Rmnd5a	Required for mitotic nuclear division 5 homologue A (S. cerevisiae)	1.32	.000062927
Rnf114	Ring finger protein 114	1.32	.000214415
Krtap31‐2	Keratin associated protein 31–2	1.32	.000095722
Gpr17	G protein coupled receptor 17	1.32	.000322515
Otx2	Orthodenticle homologue 2 (Drosophila)	1.34	.000045170
Ucp2	Uncoupling protein 2 (mitochondrial, proton carrier)	1.34	.000467815
Plp1	Proteolipid protein (myelin) 1	1.35	.000000428
Snord116	Small nucleolar RNA, C/D box 116	1.35	.000004984
Mir382	microRNA 382	1.4	.000372021
CD24a	CD24a antigen	1.4	.000069054
Cbln2	Cerebellin 2 precursor protein	1.41	.000079102
Taf1d	TATA box binding protein (Tbp)‐associated factor, RNA polymerase I, D	1.43	.000002259
Igf2	Insulin‐like growth factor 2	1.45	.000002819
Txnip	Thiredoxin interacting protein	1.52	.000373164
Rny3	RNA, Y3 small cytoplasmic (associated with Ro protein)	1.53	.000183736

To assess whether specific gene ontologies or pathways were overrepresented within the list of genes differentially expressed after enucleation we performed gene ontology (GO) analysis using DAVID (Huang da et al., 2009a, 2009b). GO analysis includes the cellular compartment which genes are active in, the molecular function the gene performs or the broader biological process that a gene is within. Overrepresented gene ontologies or pathways may suggest how retinal input regulates dLGN function. Twelve biological process GO terms were enriched in the differentially expressed genes. Most of them are broad categories such as “cell surface receptor linked signal transduction,” “transmission of nerve impulses,” and “regulation of kinase activity” which do not point at particular biological functions for further in‐depth analysis. Similarly, the cellular components that were identified also relate primarily to cell signaling and nerve transmission pathways as many of the genes reside in the plasma membrane and extracellular regions.

We also considered a list of genes with relative expression of > 1.25 or < 0.8, which contained a total of 80 differentially regulated genes. As before, more genes were down‐ than upregulated on the enucleated side. Of these 29 additional genes, we included some with relevant biological functions in our further analysis: Adamts3, Timp4, and Egr2. Adamts3, a metalloprotease, was chosen because it is within a family of genes that regulate chondroitin sulfate proteoglycans (including aggrecan). Timp4 is within a family of metalloprotease inhibitors that regulate Adamts metalloproteases (Kashiwagi et al., 2001). As such both were deemed biologically relevant, given the previous evidence for the involvement of aggrecan in layer 6 and 6b ingrowth to the dLGN and cortical plasticity after sensory deprivation (Brooks et al., 2013; Grant et al., 2016; Kind et al., 2013; Matthews et al., 2002; McRae et al., 2007). Egr2 is an immediate early gene which has been shown to be altered following MoE in the cortex (Kaczmarek & Chaudhuri, 1997; Majdan & Shatz, 2006; Nys et al., 2014; Van Brussel et al., 2011).

Microarray analysis of P3 dLGN and LP under control and enucleation conditions (Frangeul et al., 2016) had revealed an identity shift toward a gene expression pattern in the enucleated dLGN more similar to higher order thalamic nuclei.

### 3.2 qPCR validation of differentially expressed genes

To validate the microarray results, 22 genes were chosen from the microarray list of differentially expressed genes to perform RT qPCR. Eight genes, which were expressed at > 1.5 or < 0.66 after enucleation were included for qPCR validation. RIKEN cDNA E530001K10 and Rny3 were the only genes with a relative expression of > 1.5 or < 0.66 not included as commercially available primers did not exist.

Ten genes from those with a relative expression of > 1.3 or < 0.77 were chosen for qPCR verification. These were chosen from the larger list by biological relevance. They were Vsnl1, Myot, Moxd1, Kcnn3, Igf1, Calb2, Otx2, CD24a, Cbln2 Gjd2, and Igf2. Additionally, Adamts3, Timp4, and Egr2 were included to confirm that they were not false positives as they were outside the < 0.77 threshold.

**FIGURE 1:**
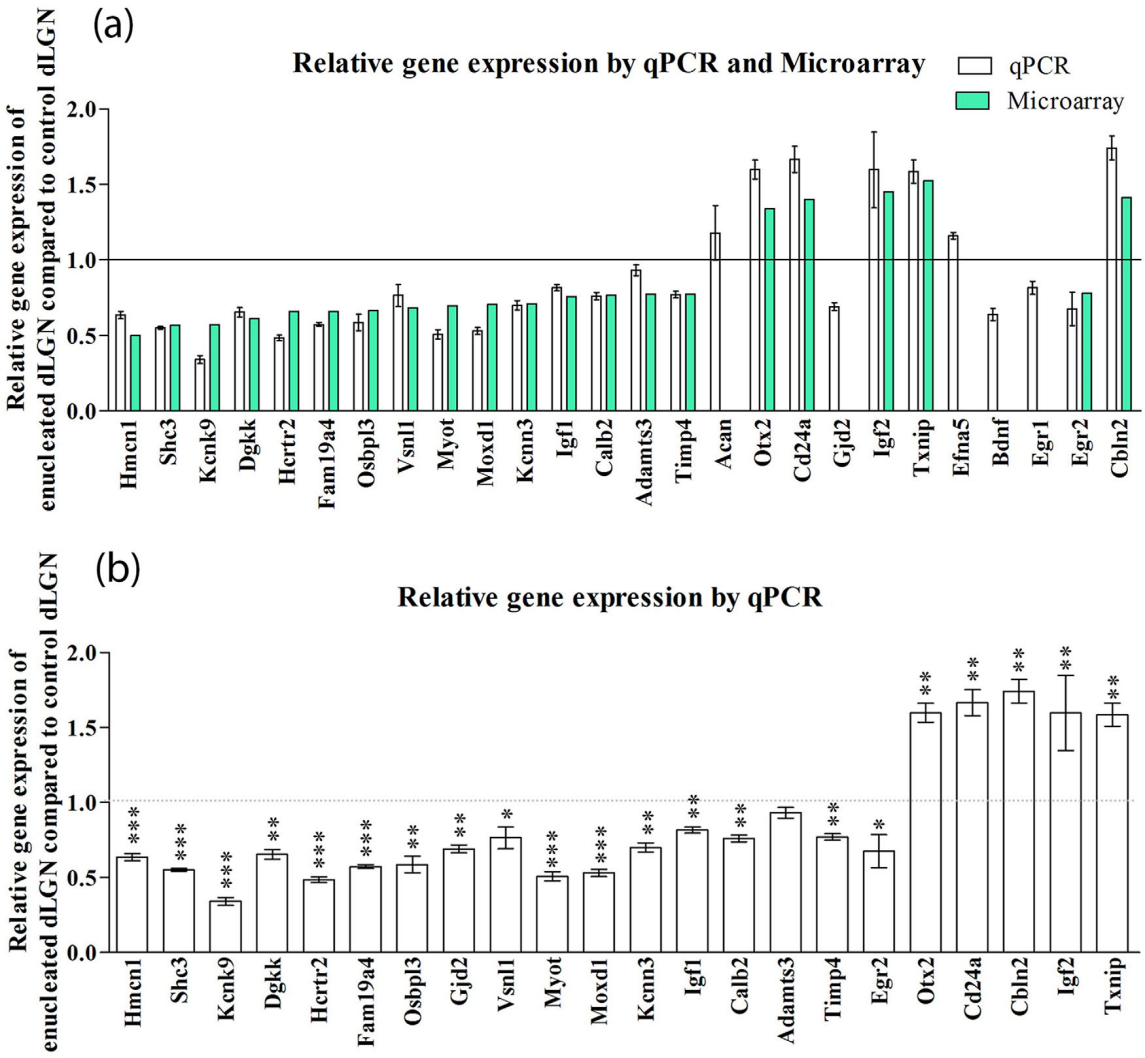
Fold change of gene expression in enucleated dLGN (contralateral to the enucleation) compared to control dLGN (ipsilateral to the enucleation) for the qPCR validation of the microarray results. (a) 22 genes were chosen from the microarray list of differentially expressed genes to perform real time, quantitative PCR (RT qPCR). Relative expression of all the genes, as assessed by qPCR, was in the same direction as relative expression assessed by the microarray. (b) The significance of relative gene expression level of 22 genes in the enucleated dLGN compared to the control dLGN, as assessed by qPCR. All but one of these 22 genes (Adamts3) were significantly differently expressed in the enucleated dLGN comprared to the control dLGN. Values shown are mean and standard error. One tailed, paired *t*‐test was performed to assess statistical significance. * = significant at *p* = .05, ** = significant at *p* = .005, *** = significant at *p* < .0005. dLGN, dorsal lateral geniculate nucleus

All but one of these 22 genes were significantly differently expressed between enucleated and control dLGN with qPCR (Figure 1b). All genes showed differential expression in the same direction as shown by the microarray (Figure 1a). Only Adamts3 was not significantly differentially expressed as assessed by this qPCR (Figure 1b).

### 3.3 Gene expression changes after enucleation compared with normal gene expression in different thalamic nuclei

Of the 51 genes identified by the more stringent microarray analysis, only nine had developmental expression patterns available on the Allen Brain Atlas Developing Mouse brain. Thus, we used the adult in situ hybridization data on the Allen Mouse Brain Atlas to determine the thalamic expression pattern. Forty‐two of the 51 differentially expressed genes had in situ hybridization data available (82%), although many were only weakly, and more or less uniformly expressed in the thalamus. Of the 33 genes whose expression was downregulated on the enucleated side, 12 genes were differentially expressed across dorsal thalamus in adult brains. Specifically, eight genes were more strongly expressed in dLGN than the adjacent, higher order LP (24%; Fam19a4, Dusp4, Gfra1, Chst2, Pdlim5, Fos, Adra1d, Gjd2), one was strongly expressed in LP but not dLGN (3%; Calb2) and another one showed strong expression in vLGN, but neither dLGN nor LP (3%; Chrm2). Conversely, of the 14 genes whose expression was upregulated on the enucleated side, two genes were more strongly expressed in dLGN than LP (14%; Mxd4, CD24a), two genes were more strongly expressed in LP than dLGN (14%; Igf2, Cbln2), one of which also fell into the category of more strongly expressed in vLGN than either of dLGN or LP (14%; Otx2, Cbln2). Thus, genes that show stronger expression in dLGN compared to LP in adult brains were enriched among the genes downregulated in dLGN following MoE.

### 3.4 Comparison of gene expression changes after enucleation with normal developmental trajectory

**FIGURE 2:**
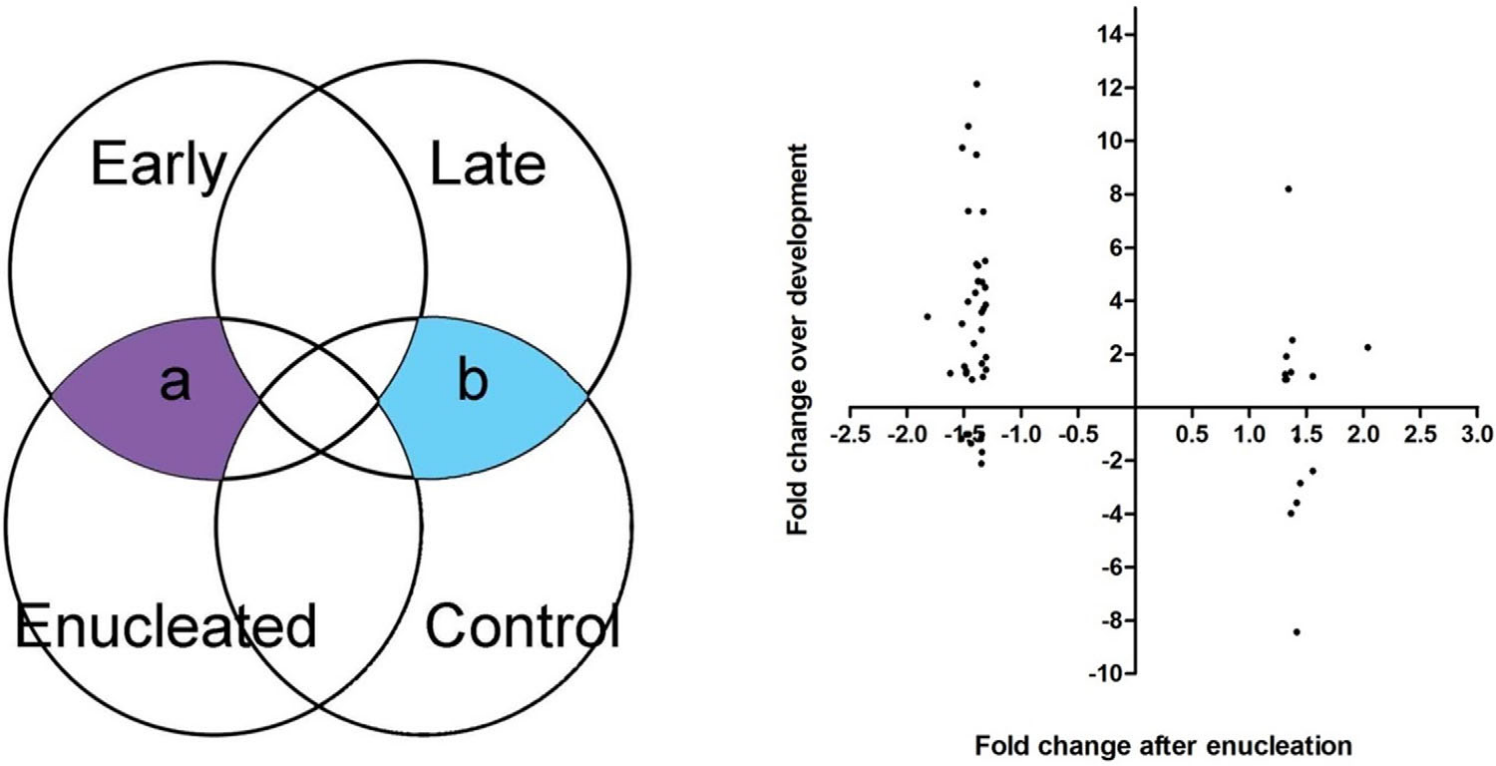
Delay in dLGN transcriptome maturation after P0 enucleation. The schematic left panel indicates the comparisons of our dLGN gene expression data of enucleated and control dLGN from monocularly enucleated mice at P6 with the normal dLGN gene expression at P0 and P10 from Frangeul et al. (2016). Right panel: Graph showing genes which were differentially expressed after enucleation and over development. The fold change of genes differentially expressed in the enucleated dLGN compared to the control dLGN was plotted against their fold change value in the P10 dLGN compared to the P0 dLGN. Most genes are downregulated in the enucleated dLGN. Most of these downregulated genes are upregulated in the dLGN over development. Of the few genes which were upregulated after enucleation, eight were upregulated and six were down regulated over development. The transcriptome of P0 enucleated dLGN at P6 is more similar to the early dLGN gene expression (a) than to the age‐matched controls (b) suggesting delayed transcriptomic maturation. The normal developmental data has been compared to the gene expression changes observed in the enucleated dLGN. Left panel depicts the fold change after enucleation plotted against fold change over development

We chose to systematically investigate whether genes whose expression is altered after enucleation are also developmentally regulated. Therefore, we compared our microarray results with the results from a microarray relating the dLGN at P0 and P10 microarray analysis performed in the laboratory of Professor Denis Jabaudon (University of Geneva). This microarray was performed on the Affymetrix GeneChip Mouse Genome 430 2.0 Array and compared gene expression in the dLGN at P0 and P10 (Frangeul et al., 2016). There are four potential expression profiles that a gene could show after enucleation and over development: (1) Genes whose differential expression after enucleation is premature and would occur in the same direction over development. (2) Genes whose normal developmental regulation is delayed or disrupted by loss of input and as such would change in one direction after enucleation and change in the opposite direction over development. (3) Genes whose expression is altered after MoE but is not normally developmentally regulated. (4) Genes whose expression is developmentally regulated but are not affected by MoE. To perform this comparison our control and enucleated .CEL files were analyzed using the same statistical method as used to analzse the developmental array for compatibility (ANOVA and *t*‐test intercept method). This analysis identified genes which were differentially expressed both between control and enucleated dLGN at P6, and between the P0 control dLGN and the P10 control dLGN (table in Figure 2). Direct microarray comparison could not be performed because different microarray chips were used. With a fold change cutoff of greater than 1.3 (or −1.3, equivalent to < .77 relative expressions) 69 genes were differentially expressed between the enucleated and the control dLGN. Forty‐two were downregulated and 26 were upregulated in the enucleated dLGN. Forty‐three genes which were differentially expressed after enucleation were also differentially expressed at P10 compared P0 (Table 2 and Figure 2). Fold change (rather than relative expression) was used to tabulate and graph these results to allow comparison with Frangeul et al. (2016). Nineteen genes were identified in the ANOVA/*t*‐test intercept analysis method but were not identified by the Limma analysis method (asterisk "*" in table in Figure 2). These genes were discarded from further analysis because the Limma multiple testing correction is thought to be the most robust for microarray bioinformatics analysis thus reducing the chance of false positives (Smyth, 2004). Of the 43 genes which were differentially expressed both after enucleation and over development, 30 were downregulated in the enucleated dLGN, 13 were upregulated in the enucleated dLGN (table in Figure 2).

**TABLE 2:** List of the genes with differential expression in the enucleated dLGN compared to the control dLGN (left) and the P10 dLGN compared to the P0 dLGN (right). List generated using ANOVA/*t*‐test intercept method using a fold change cut off value of > 1.3 or ← 1.3. Downregulated genes are shaded blue. Upregulated genes are shaded red. Genes not identified in Limma analysis denoted*

Gene symbol	Gene name	Fold‐change	Fold‐change over development P0‐P10
Shc3	src homology 2 domain‐containing transforming protein C3	−1.8219	3.4082
Hcrtr2	hypocretin (orexin) receptor 2	−1.62088	1.2800
Osbpl3	oxysterol binding protein‐like 3	−1.52345	−1.1790
Moxd1	monooxygenase, DBH‐like 1	−1.51864	3.1421
Myot	myotilin	−1.51491	9.7488
Tacstd2	tumor‐associated calcium signal transducer 2	−1.49733	1.5370
Spred2	sprouty‐related, EVH1 domain containing 2	−1.48255	1.2718
Gjd2	gap junction protein, delta 2	−1.47835	1.3583
Fam19a4	family with sequence similarity 19, member A4	−1.4642	3.9706
Vsnl1	visinin‐like 1	−1.46326	10.5566
Kcnn3	potassium intermediate/small conductance calcium‐activated channel, subfamily N, member 3	−1.45767	−1.0089
Ecm2	extracellular matrix protein 2	−1.42854	1.0527
Ptgr1	prostaglandin reductase 1	−1.41455	2.3981
Sncg	synuclein, gamma	−1.40086	4.3147
Synm	synemin, intermediate filament protein	−1.39202	9.4875
Dgkg	diacylglycerol kinase, gamma	−1.38908	12.1429
Pdlim5	PDZ and LIM domain 5	−1.37741	5.3178
Egr2	early growth response 2	−1.35228	−1.1871
	2610318N02Rik *	−1.34751	−2.1116
Shisa6	shisa homologue 6 (Xenopus laevis)	−1.34632	3.5749
Fos	FBJ osteosarcoma oncogene	−1.34455	1.6558
Hmgb2	high mobility group box 2 *	−1.34431	−1.6807
Calb2	calbindin 2	−1.34087	4.7060
Lce1c	late cornified envelope 1C *	−1.33336	1.1469
Igf1	insulin‐like growth factor 1	−1.33168	7.3543
Pvt1	plasmacytoma variant translocation 1 *	−1.3302	3.7026
Timp4	tissue inhibitor of metalloproteinase 4	−1.31561	5.5110
Hmgn5	high mobility group box 5	−1.30998	3.8549
Chst2	carbohydrate sulfotransferase 2	−1.3083	1.4138
Gfra1	glial cell line derived neurotrophic factor family receptor alpha 1	−1.30756	1.8884
Ucp2	uncoupling protein 2	1.31643	1.2418
Tac1	tachykinin 1 *	1.3246	1.9175
Rnf114	ring finger protein 114	1.32678	1.0594
Gpr17	G protein‐coupled receptor 17	1.34438	8.1938
Hist1h4c	histone cluster 1 h4c *	1.36685	−3.9876
Rmnd5a	required for meiotic nuclear division 5 homologue A	1.36687	1.3232
Otx2	orthodenticle homologue 2 (Drosophila)	1.37712	2.5278
Cd24a	CD24a antigen	1.41627	−8.4301
Taf1d	TATA box binding protein (Tbp)‐associated factor, RNA polymerase I, D	1.4188	−1.2019
Cbln2	cerebellin 2 precursor protein	1.44633	−2.8478
Txnip	thioredoxin interacting protein	1.55786	−2.3892
Ptgds	prostaglandin synthase *	2.03973	2.2592

The results of Figure 2 and Table 2 demonstrate that, while the majority of genes are downregulated in the enucleated dLGN compared to the control dLGN, the majority of those genes (25/30) are normally upregulated between P0 and P10. Of the genes which were upregulated after enucleation there was not a trend in direction of changing expression over development: seven were upregulated over development, six were downregulated over development (Figure 2 and Table 2). Thus 12 genes were changed in the same direction after enucleation and over development whereas 30 genes were changed in the opposite direction after enucleation as over development. This suggests that loss of retinal input to the dLGN prevents the normal maturation of the transcriptome of the dLGN relay neurons. These results suggest that the enucleated dLGN is transcriptionally delayed compared to the control dLGN.

### 3.5 Changes in gene expression confirmed with in situ hybridization for selected genes

We selected four genes from the 22 qPCR validated list, for further expression analysis by in situ hybridization. We selected *Otx2*, *Efna5*, *Calb2*, and *Cbln2* based on their different molecular functions. All in situ hybridization was performed on coronal sections of P8 mouse brains, where pups had undergone MoE at P0.

**FIGURE 3:**
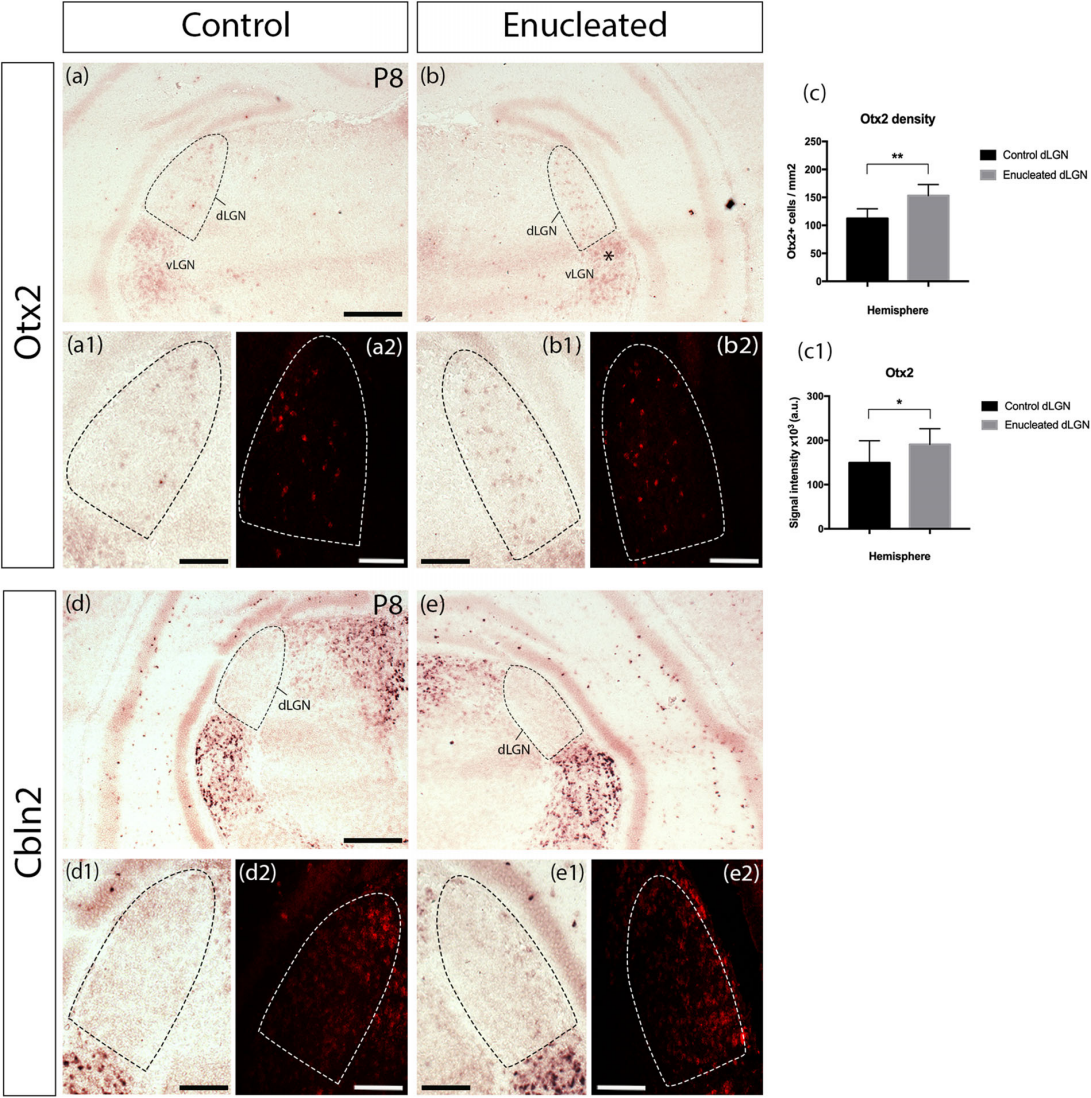
Comparisons of gene expression patterns after neonatal monocular enucleation between the control (ipsilateral to the enucleation) and enucleated (contralateral to the enucleation) sides of the same brain for selected genes revealed with in situ hybridization at P8. Low power permanent (upper panels) and higher power permanent and fluorescent (lower panels) in situ hybridization images of selected gene expression patterns in dLGN and vLGN at P8 ipsilateral to (control, left columns) and contralateral to (enucleated, right columns) the neonatal enucleation. We show two selected gene expression patterns: *Otx2* (a–c1) and Cerebellin 2 precursor protein *Cbln2* (d–e2). Cell density (c) and signal intensity (c1) of *Otx2* were significantly increased in the enucleated dLGN compared to the control. In addition to the changes of dLGN gene expression after enucleation, we observed increased expression of *Otx2* in the vLGN of the enucleated side. Results for quantifications for (c), (c1), (f), (i), and (l) based on *n* = 3 animals, at least three medial sections of control and enucleated dLGN per animal. Values shown are mean and standard error. ** = significant at *p* < .01, **** = significant at *p* < .0001. dLGN, dorsal lateral geniculate nucleus; vLGN, ventral lateral geniculate nucleus; a.u., arbitrary unit. Scale bars: 200 μm

Otx2 is a transcription factor, which is considered essential for the regulation of interneuron development, migration, and plasticity in the visual system. It is expressed specifically by dLGN GABAergic interneurons and not dLGN thalamocortical neurons. Otx2 expression has been documented in a subpopulation of interneurons in the ventral LGN (vLGN), indicating common origins with the interneurons expressing *Otx2* in dLGN (Golding et al., 2014; Sugiyama et al., 2008). In the microarray, *Otx2* expression was upregulated in the enucleated dLGN, and this change was confirmed by qPCR. Further in situ hybridization against *Otx2* revealed a few, distinctly labeled cells on the control side dLGN (Figure 3a‐a2) compared to cells occupying the majority of dLGN on the enucleated side (Figure 3b‐b2). Density of DAPI+ Otx2+ cells was significantly increased in the enucleated dLGN (Figure 3c, *p* = .0087, two‐tailed, paired Student's *t*‐test), as well as signal intensity (Figure 3c1), *p* = .0408, two‐tailed, paired Student's *t*‐test) compared to control hemisphere. Moreover, an upregulation in the vLGN expression of Otx2 in the enucleated side was observed (Figure 3a,b).

Cerebellin 2 precursor protein (Cbln2) is a synaptic organizer localized in Purkinje cells and plays a role in synaptogenesis. During normal development in the mouse dLGN, *Cbln2* expression is downregulated between P3 and P8 (Singh et al., 2012). In the microarray and qPCR, *Cbln2* expression was upregulated in the enucleated dLGN compared to the control. Our own in situ hybridization experiments confirmed that *Cbln2* is more strongly expressed in vLGN and dLGN, and somewhat more strongly expressed in LP compared to dLGN (Figure 3d‐d2, e‐e2).

**FIGURE 4:**
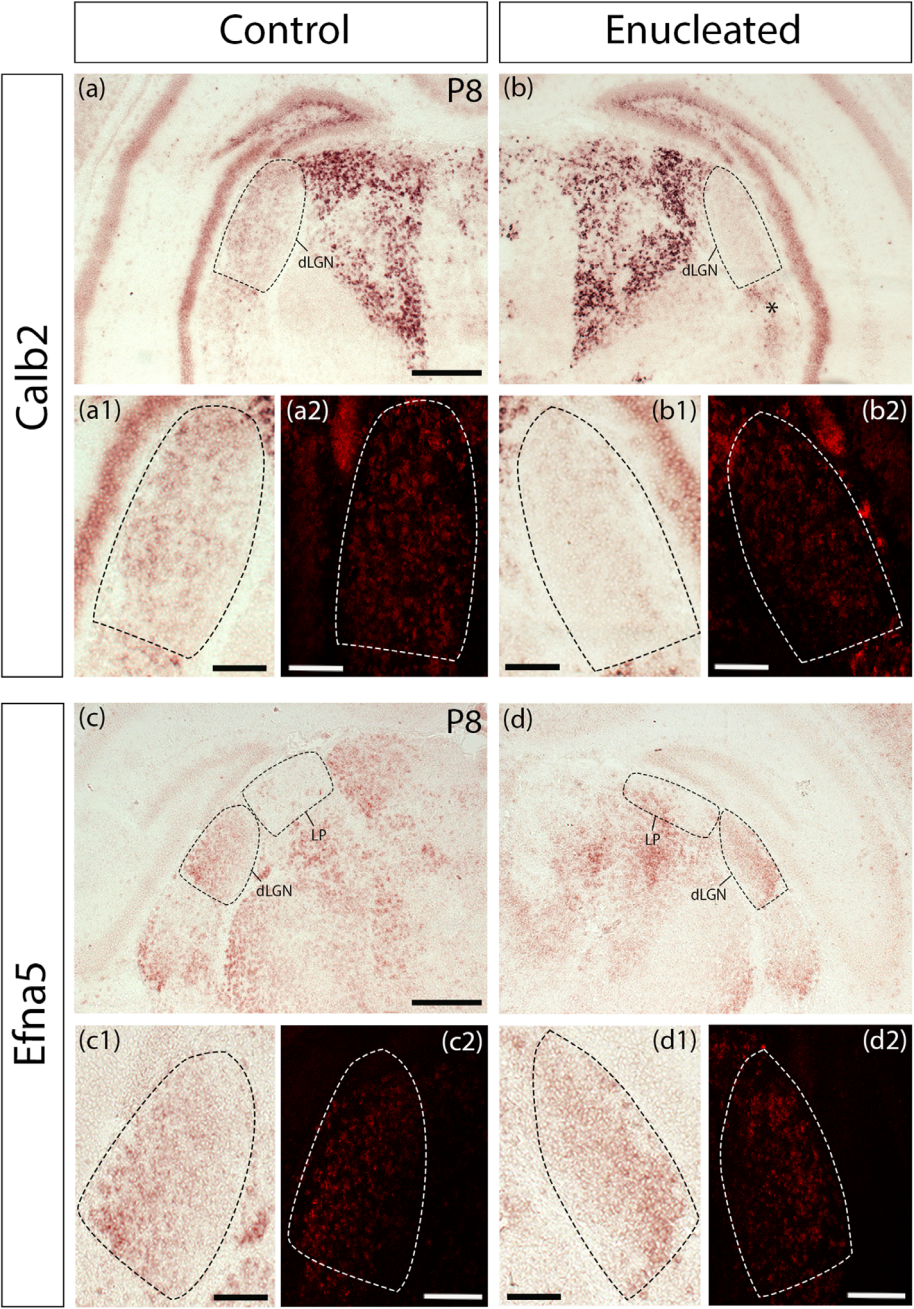
Comparisons of gene expression patterns after neonatal monocular enucleation between the control (ipsilateral to the enucleation) and enucleated (contralateral to the enucleation) sides of the same brain for selected genes revealed with in situ hybridization at P8. Low power permanent (upper panels) and higher power permanent and fluorescent (lower panels) in situ hybridization images of selected gene expression patterns in dLGN, vLGN, and LP at P8 ipsilateral to (control, left columns) and contralateral to (enucleated, right columns) the neonatal enucleation. We show two selected gene expression patterns: Calbindin 2 (*Calb2*) (a–b2) and *Efna5* (c–d2). *Calb2* expression appeared to be downregulated after enucleation (b1‐b2). In addition to the changes of dLGN gene expression after enucleation, we observed additional modifications in other thalamic nuclei. We detected increased expression of *Calb2* in the vLGN of the enucleated side, as well as increased *Efna5* expression in the enucleated LP. Abbreviations: dLGN, dorsal lateral geniculate nucleus; vLGN, ventral lateral geniculate nucleus; LP, lateral posterior nucleus. Scale bars: 200μm

*Calbindin 2* (*Calb2*) encodes the calcium‐binding protein calretinin, which is expressed in retinal axons projecting to the dLGN (Su et al., 2011). During normal brain development, *Calb2* expression in dLGN increases between E18.5 and P14 based on in situ hybridization images from the Allen Brain Atlas Developing Mouse brain. *Calb2* was found to be downregulated in the deprived dLGN in our microarray, and developmentally shows stronger expression in vLGN and LP than in dLGN at P4, but more uniform expression in LP and dLGN by P14. Across the entire structure of dLGN, the intensity of in situ hybridization signal is weaker on the enucleated side compared with the control dLGN (Figure 4a‐a2, b‐b2).

*Efna5* is a cell surface‐bound ligand for the Eph receptor family. In the thalamocortical system, *Efna5* has been characterized for its repellent activity for somatosensory thalamocortical axons that express Eph receptors. Additionally, it was shown to play a role in the regulation and specificity of the topography of thalamocortical projections within specific cortical areas, including retinotopy in the visual system (Dufour et al., 2003; Vanderhaeghen et al., 2000). In the molecular analysis performed previously in our laboratory, *Efna5* was selected out of biological interest for validation of its expression in dLGN by qPCR following MoE. The results of the qPCR revealed a significant increase in the expression of this gene in the deprived dLGN at P6. This result is in contrast with a previous study by Dye et al. (2012), where it was shown by in situ hybridization that the area of expression of Efna5 was decreased in dLGN after enucleation (Dye et al., 2012). Our in situ hybridization validates the qPCR results as the expression of *Efna5* showed stronger expression and maintained the lateral (strong) to medial (weak) gradient of expression across dLGN (Figure 4c‐c2, d‐d2). However, on the control side, *Efna5* expression was virtually absent from LP (Figure 4c), whereas LP on the enucleated side contained a dense cluster of *Efna5* labelling (Figure 4d).

### 3.6 Confirming regional source of aberrant cortical fibers in dLGN and evidence for synapse formation

The microarray results and selected gene expression changes after MoE, hinted at a change of molecular identity, with changes in genes differentially expressed between dLGN and LP being particularly affected by MoE. Assessing Allen Brain Atlas adult mouse brain in situ hybridization data did not suggest any switch of sensory modality, that is, gene expression altered by MoE was not enriched for genes enriched in other primary sensory thalamic nuclei such as VB. To assess whether enucleated dLGN might change areal identity, for example, increase in similarity to VB, for example, we examined whether the source of the aberrant cortical fibers innervating enucleated dLGN is from S1.

**FIGURE 5:**
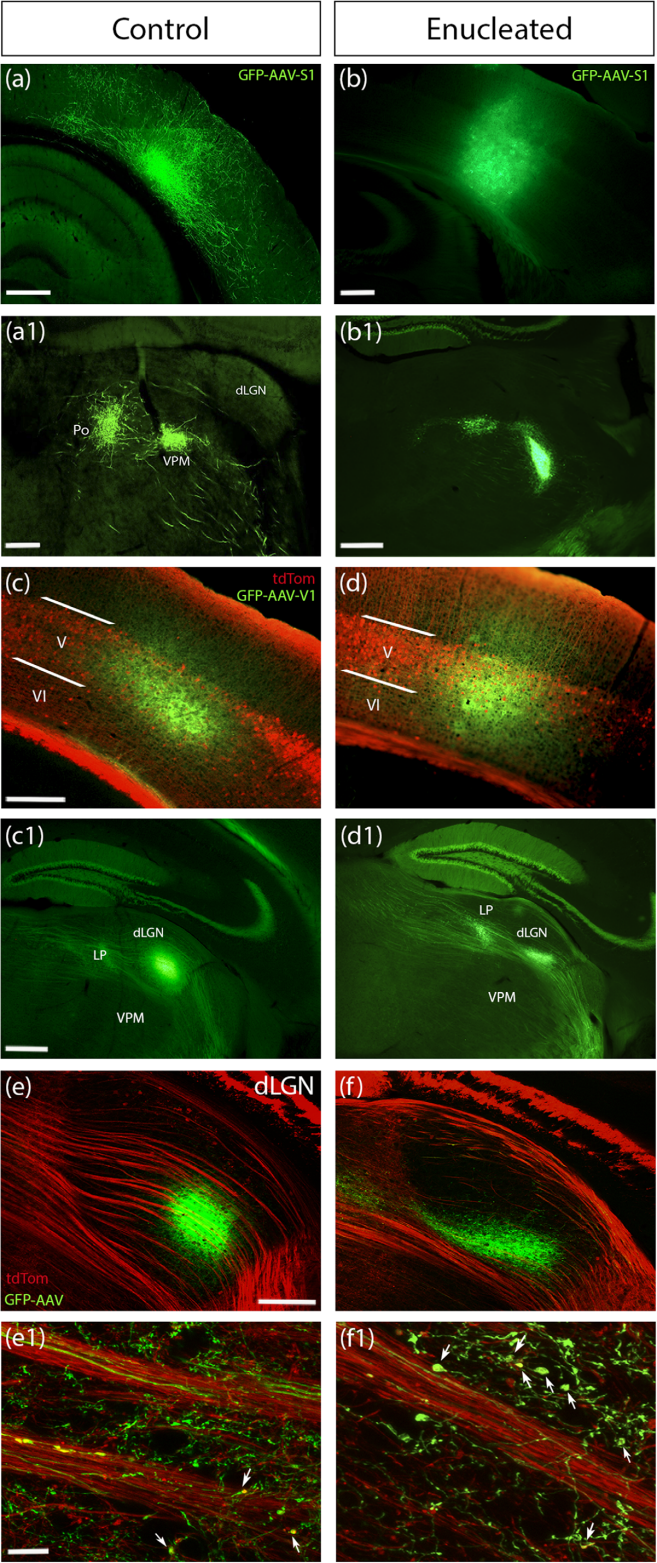
Layer 5 corticothalamic axons originate from V1 and not S1 in the contralateral dLGN following monocular enucleation at P0 in adult Rbp4::tdTom mice. Coronal sections of cortical S1 and V1 injections with AAV GFP Cre‐independent virus in adult mice. (a‐a1) In control conditions (no enucleation), corticothalamic projections from S1 innervate the somatosensory thalamic nuclei, VPM and Po (images adapted from Allen Institute for Brain Science). (b‐b1) In mice monocularly enucleated at P0, corticothalamic projections follow the same pattern as in the control non enucleated condition, innervating only the VPM and Po and completely bypassing dLGN. (c, d) High magnification images showing the colocalization of GFP+ tdTom+ cells at the site of injection in V1. Note that the majority of the GFP+ cells are situated in layer 5 colocalizing with layer 5 tdTom+ cells, with only low GFP signal detected outside of layer 5. (c1, e‐e1) In control mice, corticothalamic axons from V1 project to the visual thalamic nuclei, dLGN and LP. (d1, f‐f1) In monocularly enucleated mice, axons from V1 innervate contralateral (enucleated) dLGN and LP, showing rewiring inside dLGN compared to the control mice, with axons crossing through the lower latero‐medial part and the dorsal part of the structure. (e1, f1) High magnification images of dLGN with tdTom+ GFP+ axonal terminals (white arrows) in control and enucleated dLGN, respectively. (b1), (c1), and (d1) images have faint green signals in hippocampal mossy fibers and in dentate gyrus because of slight bleed‐through of the very strong tdTomato signal. Abbreviations: AAV, adeno‐associated virus; GFP, green fluorescent protein; S1, primary somatosensory cortex; V1, primary visual cortex; VPM, ventral‐posteromedial nucleus; Po, posterior thalamic nucleus; dLGN, dorsal lateral geniculate nucleus; LP, lateral posterior nucleus; V, layer 5; VI, layer 6. Scale bars: (a‐a1) were scaled as (b–f1), 200 μm (b‐c1, e, f), 10 μm (e1, f1)

It has previously been reported in the literature, that enucleated dLGN is prematurely innervated by cortical layer 6a fibers (Brooks et al., 2013; Grant et al., 2016), as well as aberrantly innervated by cortical L5 fibers, that would normally arborize and synapse in the adjacent higher order LP nucleus (Frangeul et al., 2016; Grant et al., 2016). But the cortical area from which these layer 5 axons derive had not been assessed at the time. Subsequently, Frangeul et al. (2016) demonstrated functional synaptic connectivity between Rbp4‐Cre expressing L5b neurons in primary visual cortex and dLGN neurons after binocular enucleation. We used injection of GFP expressing AAV into L5 of primary somatosensory and primary visual cortex in Rbp4‐Cre;Ai14 adult mice that had undergone MoE at birth, to further probe whether some of the aberrant L5 innervation in enucleated dLGN could be derived from cross‐modal (from different cortical area), rather than cross‐hierarchical (layer 5 from primary visual cortex) rewiring. The virus used for these injections was not Cre‐dependent. Our injections were aimed at L5 and most labeled cell bodies were located in L5, but some additional L6 cells and their processes are also likely to be labeled in our experiments (Figure 5c,d).

S1 afferents are well known to avoid dLGN under control conditions (Allen Institute for Brain Science, 2013). Our injections of GFP‐expressing virus into S1 of neonatally enucleated mice did not label any GFP+ afferents in dLGN on the enucleated side, (Figure 5b1), either, ruling out a contribution of cross‐modal plasticity. GFP+ afferents labeled by V1 injections were abundant in the enucleated dLGN, as well as LP, as expected. More specifically, in control conditions, axons were found to pass through the medial part of the dLGN, exhibiting a wide distribution through the center of the structure (Figure 5c1, e‐e1). However, on the enucleated side, GFP‐positive axons were present in the lower ventro‐medial part of the deprived dLGN with few axons detected to sprout through the dorsal part of the structure and colocalize with the tdTomato rewired fibers (Figures 5d1, f‐f1). In both hemispheres, axons passed through dLGN and eventually reached LP, and more specifically laterorostral lateral posterior nucleus (LPLR), as expected, showing no difference in the pattern of expression inside this structure (Figure 5c1, d1).

Overall, this suggests that layer 5 axons from cortex innervate the enucleated dLGN in a modality specific, but cross‐hierarchical pattern.

### 3.7 Induced cortical layer 5 innervation to dLGN persists into adulthood

In order to study the role of retinal input in the ingrowth of corticothalamic fibers to dLGN as well as the plastic changes occurring in the visual thalamic nuclei, the first‐order dLGN, and the higher order LP, we performed MoE at P0 to Rbp4‐Cre;Ai14 mice, in which a subset of cortical layer 5 neurons express tdTomato from before birth (Grant et al., 2016).

**FIGURE 6:**
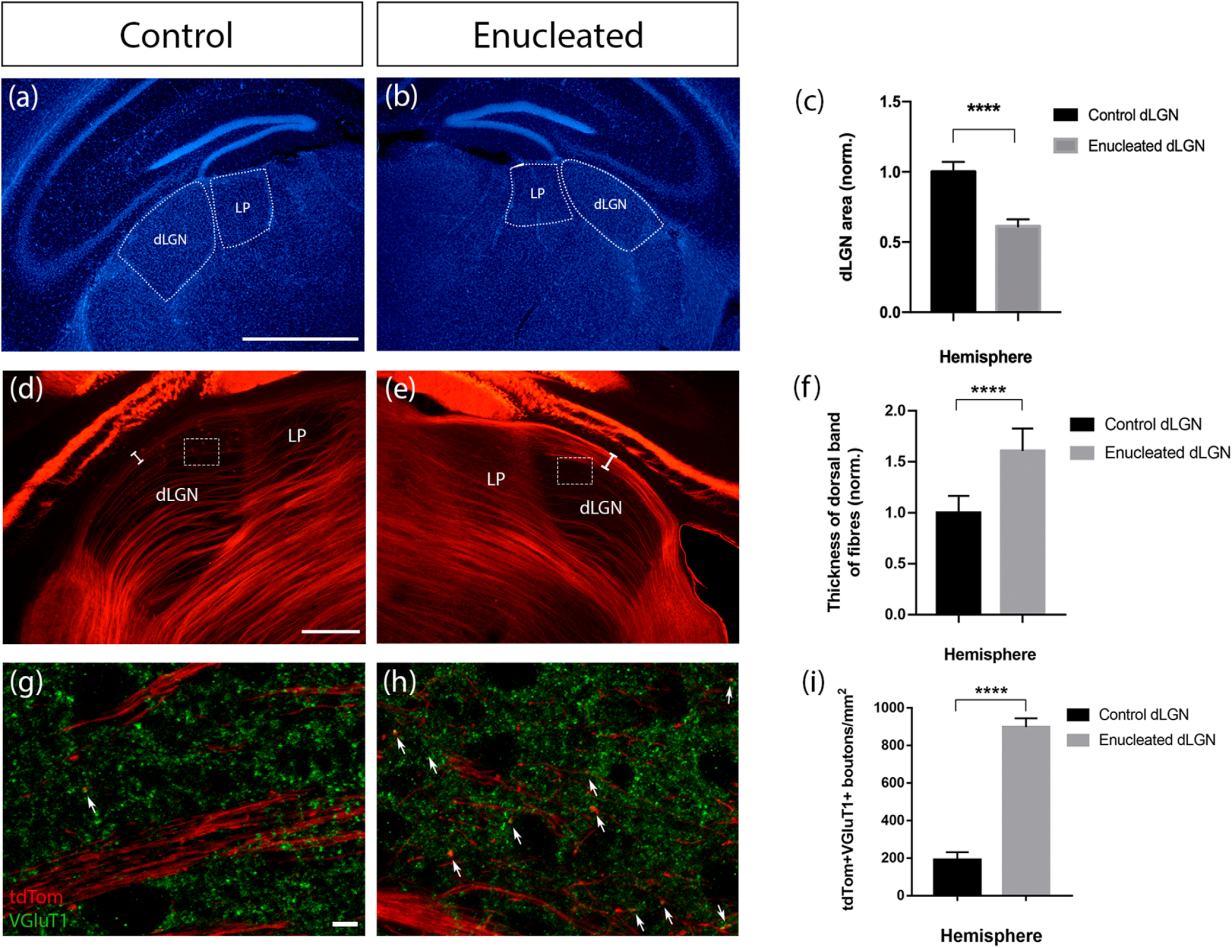
Changes in dLGN size and layer 5 axon innervation and synaptic formation in the dLGN of control (non‐enucleated mice) and monocularly enucleated Rbp4‐Cre::tdTomato adult mice contralateral to the enucleation. (a, b) The area of dLGN in mm^2^ in coronal sections in control and enucleated adult animals. After enucleation, the contralateral dLGN with reduced retinal input appears significantly decreased in size (c) in comparison with the control conditions without anucleation (area of dLGN and LP demarcated with the dotted white lines). (d) In control dLGN, layer 5 axons innervate and pass through the medial part of dLGN with very few axons crossing through the dorsal part (white bracket). (e) Following enucleation at birth, axons exhibit rearrangement inside dLGN, with projections sprouting through the dorsal part of dLGN forming a bundle of fibers (white bracket), that is significantly thicker in the enucleated dLGN (f) in comparison with the control (e). (g–h) High magnification images of the area demarcated by the white boxes in (d) and (e) respectively, demonstrating immunostaining against the presynaptic marker VGluT1, with density of synaptic boutons significantly increased in the enucleated dLGN compared to the control (i). Results for quantifications for (c) and (f) based on *n *= 9 independent samples and for (i) based on *n* = 3 animals, at least three medial sections per animal. Values shown are mean and standard error. ** = significant at *p* < .01, **** = significant at *p* < .0001. dLGN, dorsal lateral geniculate nucleus; LP, lateral posterior nucleus; VGluT1, vesicular glutamate transporter 1. Scale bars: 300 μm (a–b), 200 μm (d–e), 10 μm (g–h)

MoE at birth results in significantly reduced dLGN size on the affected side compared to the control brains where no enucleation was performed (Figure 6a–c) or compared to the contralateral dLGN of the same brains (data not shown). This reduction in size in both comparisons to controls and contralateral dLGN persisted into adulthood in our sample (compared to control 38% decrease in area, *n* = 9, *p* < .0001, two‐tailed, unpaired Student's *t*‐test). After MoE at birth, it has been reported that the bundle of cortical layer 5 axons coursing at the dorsal edge of dLGN is increased in thickness at P6 (Grant et al., 2016). This increase in axon bundle thickness following neonatal MoE also persists into adulthood (compared to control, *n* = 9, *p *< .0001, two‐tailed, unpaired Student's *t*‐test), with fewer axons visible in the centre of the enucleated dLGN, indicating a possible rearrangement of these axons into the dorsal part of the structure (Figure 6d–f).

Cortical layer 5 axons form synapses in the enucleated dLGN (Frangeul et al., 2016; Grant et al., 2016), which also persist into adulthood. Using immunostaining against VGluT1, the vesicular glutamate transporter used by cortical afferents, we identified tdTom+ VGluT1+ double‐labeled synapses in the dLGN of control and enucleated animals (Figure 6g–h). These were rare in the controls (1.92 ± 0.23 boutons/10,000 μm^2^), but significantly more common in the enucleated dLGNs (8.98 ± 0.27 boutons/10,000 μm^2^; Figure 6g–i); *n* = 3, *p* < .0001, two‐tailed, unpaired Student's *t*‐test). We have additionally observed and quantified the density of tdTom+ VGluT1+ boutons in control and enucleated dLGN of the same brains of an enucleated animal (data not shown). The double‐labeled synapses found in the control dLGN were also rare (4.24 ± 0.8 boutons/10,000 μm^2^), but their number significantly increased in the enucleated hemisphere (9.15 ± 0.36 boutons/10,000 μm^2^; *n* = 3, *p* < .0001, two‐tailed, unpaired Student's *t*‐test). Thus, the areal identify shift from first‐order to higher order like characteristics appears to persist into adulthood, and is not just a transient alteration while the brain adapts to the inflicted sensory deprivation.

## 4 DISCUSSION

The mechanisms involved in the regulation of the development and plasticity of corticothalamic projections have been largely neglected in the past with most studies focusing on the afferents from the periphery to the subcortical structures and from the thalamus to the cortex. In this study, our aim was to identify the cortical regional origin and persistence of corticofugal projections originating from layer 5 to the dLGN after visual deprivation by MoE. Building on previously reported cross‐hierarchical plasticity in dLGN (Grant et al., 2016), we studied the effects of reducing the retinal input after MoE in the specification of contralateral dLGN functional and transcriptional profile that may underlie the induction and the maintenance of the layer 5 corticothalamic projections that remain in place until adulthood. Previous research from Frangeul et al. (2016), demonstrated that, in the absence of peripheral input by binocular enucleation at P0, optogenetic stimulation of layer 5 axons in V1 elicits postsynaptic terminals in the input‐ablated dLGN neurons. This agrees with previous anatomical data suggesting that the reduction of peripheral input results in the acquisition of HO cortical input by FO neurons, showing cross‐hierarchical rewiring in dLGN after MoE at P0 (Grant et al., 2016).

### 4.1 Molecular mechanisms that regulate the corticothalamic axon ingrowth into dLGN

To evaluate the role of peripheral input in the regulation of activity‐dependent molecular mechanisms in the visual thalamic nuclei, we performed a microarray gene expression analysis in the dLGN following MoE (Grant, 2016, and this study). Genes including *BDNF*, *Egr1*, and *Egr2*, genes involved in neuronal activity, such as *Kcnn3* and *Kcnk9*, and the kinase pathway molecule encoding *Dgkk* and *Shc3*, were detected to be differentially expressed after loss of peripheral input (see Figure 1 for microarray and qPCR data and Figure 3 and 4 for in situ hybridization patterns of selected genes). Most of these genes are normally regulated during the first postnatal weeks of development, which might indicate a delay in the maturation of the dLGN transcriptome due to absence of retinal activity.

The modality‐specific input regulation of gene expression was demonstrated in an experiment when retinal input was rewired to MGN and this changed the MGN transcriptome that now included genes that are normally expressed in the dLGN (Horng et al., 2009). More specifically, 10 genes that are dLGN‐specific were found to be upregulated in the rewired MGN at P5, including the zinc‐finger transcription factor, Zic4 (Horng et al., 2009), which is strongly enriched in dLGN and has an important role in visual pathway development (Pak et al., 2004). Transcriptomic changes to S1‐S2 L4 neurons following infraorbital nerve sectioning have been revealed by Pouchelon et al. (2014). Distinct TC inputs mediate the functional molecular features of postsynaptic L4 cortical neurons in a modality‐specific manner with only a specific subset of S1L4‐ and S2L4‐type genes being affected by VB and Po input changes (Pouchelon et al., 2014). Moreover, Moreno‐Juan and colleagues (2017) have demonstrated that very early embryonic binocular enucleation in mice at E14.5 before retinal axons reach the thalamus, not only has led to an increase in the size of the cortical barrel field in S1 at P4, but also to changes in the transcriptional profile of the VPM, the corresponding somatosensory thalamic nucleus, at P0 and P4, with the RAR‐related orphan receptor B (Rorβ), which has been previously shown to influence somatosensory cortical development (Jabaudon et al., 2012), to be found significantly increased after visual deprivation (Moreno‐Juan et al., 2017).

It has been shown that after sensory ablation of VPM by infraorbital nerve sectioning this first‐order somatosensory nucleus acquired a transcriptional profile that was more similar to that of HO nuclei, supporting the hypothesis that HO identity is a default state (Frangeul et al., 2016). According to this theory, synapses originally hold HO characteristics in both first‐order and higher order thalamic nuclei and due to peripheral input, they eventually adopt their FO characteristics; thus FO identity is subsequently acquired in an input‐dependent manner (Bishop, 1959; Butler, 2008; Frangeul et al., 2016; Horvath et al., 2022; Molnár et al., 2020). From an evolutionary point of view, all the aforementioned findings of cross‐hierarchical plasticity and rewiring support the idea that neurons with HO‐like identity might be ancestors of neurons located in FO thalamic nuclei and primary cortical areas, with the latter emerging from a pool of higher order neurons based on the connectivity, electrophysiological, and metabolic characteristics (Frangeul et al., 2016).

Genetic ablation of retinal input with the *math5‐/‐* mouse model as well as induced input deprivation by binocular enucleation at birth have shown acceleration of the timing of innervation of layer 6 axons to the dLGN (Seabrook et al., 2013). Moreover, the role of aggrecan (chondroitin sulfate proteoglycan 1), an extracellular matrix protein that belongs to the perineuronal net family, has been implicated in the timing of entrance of layer 6 axons in the dLGN, demonstrating that after loss of retinal input, aggrecan is driving layer 6 and 6b axons to prematurely enter to the dLGN (Brooks et al., 2013). These results suggested that retinal inputs might initially prevent the expression of endogenous aggrecanases by dLGN relay neurons.

In our current study, we analyzed our microarray (P6 enucleated and control dLGN) data and further investigated the expression of five genes, *Otx2*, *Kcnk9*, *Efna5*, *Calb2*, and *Cbln2*, b*y* in situ hybridization to validate the microarray and qPCR data. In addition to the changes of dLGN gene expression after enucleation, we observed additional modifications in more thalamic nuclei. We detected increased expression of *Otx2*, *Kcnk9*, and *Calb2* in the vLGN of the enucleated side, as well as increased *Efna5* expression in the enucleated LP.

### 4.2 Role of Otx2 in critical period plasticity in the dLGN

We demonstrated a significant increase in the expression of *Otx2* in the dLGN by microarray, qPCR, and in situ hybridization, with the latter also showing an increase in *Otx2*+ cells in the vLGN. This might indicate a delay of interneurons to enter the dLGN possibly due to the loss of peripheral input, thus revealing the effect of peripheral input in the timing of interneuron migration to dLGN. Specific transfer of Otx2 and BDNF to GABAergic inhibitory parvalbumin interneurons, which receive the most potent direct thalamocortical input, is essential and sufficient for the onset of the critical period of plasticity in the developing murine visual cortex by endogenously coordinating parvalbumin cell maturation (Sugiyama et al., 2008). The accumulation of Otx2 in these cells is a noncell autonomous process, with Otx2 transferred from other areas, such as the retina and dLGN, to V1 and its capture dependent upon visual input as it has been reported to be reduced in the visual cortex upon dark rearing (Sugiyama et al., 2008).

Our study revealed a highly interesting change in Efna5 expression in the visual thalamic nuclei following enucleation suggesting that Efna5 may be involved in the guidance and rewiring of corticothalamic projections. EphA‐EphrinA signaling has been shown to be involved in the establishment of cortical areas and the guidance of thalamocortical projections in the visual system (Ellsworth et al., 2005). Experiments in double knockout EphrinA2/A5 mice, in which input ablation was induced, showed that rewiring was increased by the lack of these Ephrin ligands for which retinal axons have receptors (Lyckman et al., 2001). However, just the absence of these ligands was not sufficient to induce rewiring, indicating the additional role of molecular cues in the guidance of sensory afferents to their respective thalamic areas. Therefore, intrinsic molecular cues and role of activity in the remodeling of corticothalamic and thalamocortical connections have to be considered. Additionally, alteration of the interaction of ephrinA gradients in the cortex using ephrinA2/A3/A5 knockout mice resulted in changes in the size and location of visual cortices (Cang et al., 2005), indicating that changes in the expression of this molecule might induce alterations on multiple levels of visual circuit formation. Moreover, Dye et al. (2012) have demonstrated by in situ hybridization a downregulation in the expression of Efna5 in the dLGN after binocular enucleation at birth (Dye et al., 2012), a pattern we confirmed in the P8 dLGN after P0 MoE in our study, although this does not match our qPCR results. However, the similarity in the expression of Efna5 in dLGN and LP following MoE observed in the in situ hybridization experiments could possibly indicate the role of retinal input in defining the transcriptional identity of the first‐order and higher order thalamic nuclei. This hypothesis is supported by the transcriptional analysis presented by Frangeul et al. (2016), where the genetic profile of first‐order nuclei became more similar to the one of higher order nuclei after input deprivation, suggesting that the determination of molecular identity of the different orders of thalamic nuclei is activity‐dependent. Future experiments investigating the functional relevance of the genes that are differentially expressed in the dLGN after MoE at birth are necessary for determining the mechanism and level of implication in the plasticity of corticothalamic axons that reach the dLGN. Overexpression of these genes in the enucleated dLGN by in utero electroporation to observe a blockage of the L5 ectopic sprouting, or depletion of these genes by shRNA in the control dLGN may induce L5 ectopic sprouting in the absence or reduction of retinal input alterations.

### 4.3 Corticothalamic axons originate from V1 after peripheral manipulation

Our results from the viral tracing study demonstrated that, after peripheral input deprivation by MoE, corticothalamic axons originate from V1 and not from S1. Projections originating from V1 reached the latero‐ventral part of dLGN, eventually sprouting into LP. On the other hand, no alterations in the S1 projections to the thalamus were observed following MoE, with S1 axons completely bypassing dLGN, as they do in control brains. Labeling from S1, the mixed layer 5 and 6 projections only innervated and sprouted to the somatosensory thalamic nuclei, the first‐order VPM, and the higher order Po. Projections from S1 did not innervate dLGN, which demonstrated that S1 was not the origin of layer 5 projections after retinal input deprivation. Cross‐modal rewiring was found previously in humans. In congenitally blind individuals, the visual cortex was activated by somatosensory and auditory stimuli (Cohen et al., 1997), while in congenitally deaf individuals, activation of the auditory cortex was observed in response to visual stimuli (Bavelier & Neville, 2002).

In the dorsal area of dLGN, thick bundles of tdTom+ layer 5b fibres were identified after MoE as signs of axon rearrangements inside dLGN. Virus tracing from V1 labeled very few GFP+ projections crossing through this specific area and there was little overlay with the tdTom+ fibres. Thus, it is possible that this bundle of tdTom+ fibres in the dorsal dLGN might not derive exclusively from V1. In this study, we have shown that these fibers do not originate from S1. However, there are many other possible sources for these layer 5 projections to dLGN that we did not study. They could originate from A1, or they might derive from another secondary area of the visual cortex, such as V2, indicating that the plasticity might be occurring inside the visual cortex. The latter can be supported from previous research from Pouchelon et al. (2014), where it was demonstrated that in the somatosensory cortex following input deprivation by infraorbital nerve sectioning, the S1 circuits formed acquire S2‐like properties, thus first‐order thalamic input is essential for the acquisition of associative cortical identity (Pouchelon et al., 2014). It has been shown that primary cortical areas such as V1 and S1, acquire transcriptional characteristics of their associative secondary cortical areas (V2 and S2, respectively) in the absence of thalamocortical input, indicating the importance of exteroceptive thalamic input in the differentiation of cortical areas of the same sensory modality (Chou et al., 2013; Pouchelon et al., 2014; Vue et al., 2013). Therefore, projections from A1 or other sources, such as secondary cortical areas, should also be further investigated to define the exact origin of projections after visual deprivation in different parts of dLGN and whether the plastic effects observed following MoE is regulated by multiple cortical areas.

### 4.4 Does retinal input affect synapse formation in dLGN?

Corticothalamic axons start to innervate the dLGN at P3, but the accumulation of the fibers is not complete until after P10 (Brooks et al., 2013; Grant et al., 2012; 2016; Seabrook et al., 2013). As for retinogeniculate axons, although they are present in the thalamus from E15.5 (Deck et al., 2013; Moreno‐Juan et al., 2017), they do not innervate and form functional synapses in the dLGN until P12 (Brooks et al., 2013; Grant et al., 2016; Seabrook et al., 2013). Most synapses present in dLGN arise from layer 6 corticothalamic neurons from V1, brainstem nuclei, and TRN inputs (Briggs & Usrey, 2008; Sherman & Guillery, 2002). Interestingly, retinal inputs comprise only 10% of the total amount of synapses found in the dLGN (Bickford et al., 2010; Sherman & Guillery, 2002). However, the importance of retinogeniculate axons in shaping the topography of corticothalamic axons in the dLGN has been recently shown. More specifically, when mice were monocularly enucleated at birth, layer 6 projections prematurely entered the dLGN (Seabrook et al., 2013). This early entry of corticothalamic projections was demonstrated in both Golli‐tau‐eGFP (for lower layer 6) and Ntsr1‐cre;tdTomato (layer 6) lines by Grant et al. (2016). Moreover, layer 5 corticothalamic axons entered the dLGN (Grant et al., 2016). Our study showed that after MoE, there is a significant increase in the formation of layer 5 boutons in the deprived dLGN in comparison with a dLGN in a control brain or in comparison with the dLGN of the control side of the same brain. These synapses from layer 5 projections are maintained to adulthood. The significant increase in layer 5 synaptic formation after MoE might be an outcome of cross‐hierarchical plasticity between the first‐order dLGN and the higher order LP and could indicate the general role of peripheral input in shaping thalamic and cortical circuits. Pouchelon et al. (2014) has shown a cross‐hierarchical plastic change in the somatosensory system, where genetic ablation of the first‐order VPM has induced input rewiring from the higher order Po into layer 4 of S1 (Pouchelon et al., 2014).

## 5 CONCLUSIONS

Research on the development of the corticothalamic connectivity after sensory input deprivation revealed new forms of plasticity and the neural basis of behavioural compensations. Understanding how the brain is rewired upon sensory loss is essential for unravelling the mechanisms underlying plasticity in the sensory deprived brain, thus gaining better insights into the translational investigation and possible therapeutic targets for individuals with a form of sensory deprivation. Our results highlight the importance of peripheral input in the development and plasticity of the corticothalamic connections and the regulation of the transcriptional profile of thalamic nuclei. We showed that the cross‐hierarchical corticothalamic rewiring of layer 5 cortical projections to the dLGN, that is elicited by visual deprivation at birth, is preserved until adulthood and that corticothalamic axons reaching the dLGN originate from V1 and not S1. Additionally, we described some of the molecular changes in the dLGN upon visual deprivation. Comparisons with developmental gene expression patters in dLGN suggest more immature and delayed gene expression in enucleated dLGN. These results allow us to hypothesize that early peripheral input to the thalamus contributes to the transcriptional and circuit hierarchy identity of thalamic nuclei. They also provide novel cues in the understanding of the compensatory mechanisms that the brain uses to adapt to altered peripheral inputs.

### PEER REVIEW

The peer review history for this article is available at https://publons.com/publon/10.1002/cne.25304.

## Data Availability

The raw data for generating the list of the genes with differential expression in the enucleated dLGN compared to the control dLGN at P6 using Limma with a fold change cut off value of >1.3 or <0.77 presented in Table 2 is available from Dr Sheen Lee (Wellcome Trust Integrative Physiology Initiative on Ion Channels (OXION), current e‐mail: sheena.lee@medsci.ox.ac.uk. Data for figure 1 and 2 contains comparisons between our data and the published data of Frangeul et al., (2016). Frangeul, L., Pouchelon, G., Telley, L., Lefort, S., Luscher, C. and Jabaudon, D., 2016. A cross‐modal genetic framework for the development and plasticity of sensory pathways. Nature, 538(7623), pp. 96–98. doi: 10.1038/nature19770. Original sections and material for figures 3,4,5 and 6 are available from the laboratory of Prof Zoltan Molnar.
